# Plasticity of the *cis*-Regulatory Input Function of a Gene

**DOI:** 10.1371/journal.pbio.0040045

**Published:** 2006-03-28

**Authors:** Avraham E Mayo, Yaakov Setty, Seagull Shavit, Alon Zaslaver, Uri Alon

**Affiliations:** **1**Departments of Molecular Cell Biology and Physics of Complex Systems, The Weizmann Institute of Science, Rehovot, Israel; University of California IrvineUnited States of America

## Abstract

The transcription rate of a gene is often controlled by several regulators that bind specific sites in the gene's *cis*-regulatory region. The combined effect of these regulators is described by a *cis*-regulatory input function. What determines the form of an input function, and how variable is it with respect to mutations? To address this, we employ the well-characterized *lac* operon of *Escherichia coli,* which has an elaborate input function, intermediate between Boolean AND-gate and OR-gate logic. We mapped in detail the input function of 12 variants of the *lac* promoter, each with different point mutations in the regulator binding sites, by means of accurate expression measurements from living cells. We find that even a few mutations can significantly change the input function, resulting in functions that resemble Pure AND gates, OR gates, or single-input switches. Other types of gates were not found. The variant input functions can be described in a unified manner by a mathematical model. The model also lets us predict which functions cannot be reached by point mutations. The input function that we studied thus appears to be plastic, in the sense that many of the mutations do not ruin the regulation completely but rather result in new ways to integrate the inputs.

## Abstract 2

A few point mutations in the *lac* operon of *Escherichia coli* are sufficient to change the nature of the transcriptional computation.

## Introduction

Much of the computation performed by transcription networks occurs in the DNA *cis*-regulatory region (CRR) of each gene. Most genes are regulated by multiple regulators (inputs) that bind their CRR. The way that these inputs combine to determine the rate of transcription is described by the *cis*-regulatory input function (CRIF) of the gene. Well-studied examples include input functions that govern developmental genes [ 1– 4] at specific locations and times, when certain combinations of regulators are active. The CRIFs are often described using Boolean functions such as AND- and OR-logic gates [ 4– 15], although graded [ 8, 16– 18] input functions are also known to occur.

**Figure 1:**
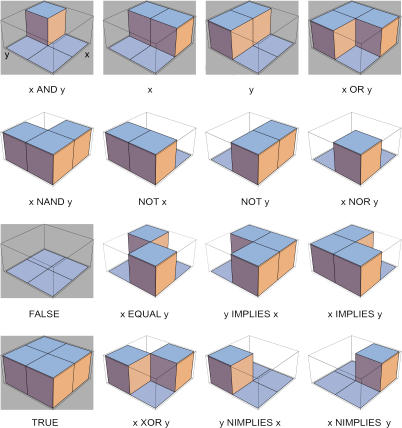
All 16 Possible Two-Input Boolean Logic Gates The gates are functions of two inputs, *x* and *y,* each of which can be 0 or 1. The top left gate, for example, is an AND gate whose output is 1 only when both *x* = 1 and *y* = 1, and zero otherwise. The AND gate is represented by a high plateau when *x* = 1 and *y* = 1, and by three low plateaus for the other combinations of *x* and *y.* The six gates with a shaded background are realizable by the model of the *lac* input function, whereas the ten gates with a white background are not (forbidden gates).

Recently, a high-resolution map of the CRIF of a well-characterized gene system, the *lac* operon [ 19– 21] of *Escherichia coli,* was obtained, using accurate gene-expression measurements from living cells [ 8]. The *lac* CRIF has two inputs, corresponding to the two regulators of the system, cAMP receptor protein (CRP) and LacI. The CRIF was found to be a rather intricate function, intermediate between Boolean AND-gate and OR-gate logic [ 8] (see Figure 1 for all 16 possible two-input Boolean logic gates). Unlike pure Boolean gates, which have two plateau levels (high and low) and one threshold per input, the *lac* CRIF has four different plateau levels and two thresholds per input [ 8].

Here, we ask which changes in a CRIF can be caused by a few point mutations in the regulatory region and which changes cannot. This question is related to the way in which the input function can be shaped by evolutionary selection [ 1]. It is believed that gene networks can “learn” new computations on an evolutionary timescale by means of mutations [ 22– 25]. Changes are mainly due to point mutations, gene duplications, and rearrangements [ 26– 28]. The degree to which mutations can change the computation, without ruining the essential function, may be termed “plasticity” [ 1, 29– 32]. The larger the plasticity, the more readily a network can learn new computations in a new environment.

To address this, we study the plasticity of the *lac* input function. We measured the effects of point mutations in the *lac* promoter region on its input function. We find that the *lac* input function is quite plastic: even a few point mutations can significantly change the CRIF, leading to input functions that resemble pure AND gates, OR gates, and single-input switches. A mathematical model explains these results and lets us predict which types of gates can and cannot be obtained with point mutations.

## Results

### Library of Variants of the *lac* CRR

**Figure 2:**
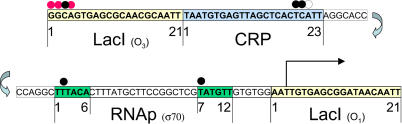
The Wild-Type *lac* Promoter Region and the Point Mutations Used in This Study Locations of point mutations are indicated by dots, where a red indicates mutations used in all variants, black means change to A or T with equal probability, and white means change to A, C, or T with equal probability. Also shown are the positions of the two LacI sites O1 and O3, the CRP site, and the RNAp site (−10 and −35 regions). Note that the promoter region is displayed in this figure in two parts: the sequence in the top part ends just downstream of the beginning of the sequence in the bottom part. The black arrow indicates the transcription start site of the *lacZ* gene.

To study the effects of point mutations in the regulator binding sites of the *lac* CRR, we constructed a random library of CRR mutants. The library was based on the 113-bp regulatory region of the *lac* operon from wild-type *E. coli.* Each CRR variant contained between three to nine point mutations in selected locations in the regulator binding sites ( Figure 2). There were at most four mutations in the O3 site of LacI, at most three mutations in the CRP binding site, and at most two in the σ-70 binding site (the binding site of RNA polymerase [RNAp]). The mutations were chosen to bring the sites significantly closer or further to the sites' consensus sequence. The CRR library was cloned into a low-copy plasmid upstream of a green fluorescent protein (GFP) gene. The plasmids were transformed into E. coli MG1655. We isolated 62 variants and measured the promoter activity in all four combinations of saturating or zero concentration of its two inducers cAMP and isopropyl-D-thiogalactoside (IPTG). Out of the 62 variants, 25 (about 40%) gave a detectable GFP signal in at least one of the four conditions. The latter were sequenced and screened for duplicate variants, yielding 12 unique variants. The CRIFs of these 12 variants were mapped in detail (see below). Note that our screen did not a prioi eliminate any potential two-input Boolean functions (e.g., XOR gates).

### Diverse Input Functions Are Generated by a Few Point Mutations

To measure the CRIF of each CRR variant, we grew the corresponding reporter strain inside a multiwell fluorimeter in defined glucose medium supplemented with 88 combinations of the two inducers cAMP and IPTG. The CRIF describes the promoter activity in the various inducer combinations. The promoter activity, which corresponds to the rate of GFP production per cell, was measured over the exponential phase of growth. The estimated experimental mean relative error based on day–day repeats was about 15%.

**Figure 3:**
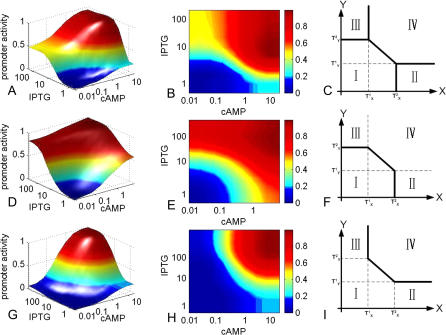
Input Function of the Wild-Type *lac* Promoter Region and Two Variant CRRs The input functions describe the promoter activity as a function of the concentrations of the two inducers IPTG (in μM) and cAMP (in mM), measured in midexponential growth in an automated fluorimeter by means of a GFP-promoter fusion on a low-copy plasmid. (A) Surface plot of log wild-type input function. (B) Contour plot of wild-type input function. (C) Schematic drawing showing the activation thresholds and the four plateaus of the input function. (D–F) Same for strain U340, with an OR-like input function. (G–I) Same for strain U339 with an AND-like input function. The plateaus of the input functions are marked by roman numerals in (C, F, and I). Plateau I occurs at zero concentration of inducers, plateau II at saturating cAMP and zero IPTG, plateau III at saturating IPTG and zero cAMP, and plateau IV occurs when both inducers are saturating.

**Figure 4:**
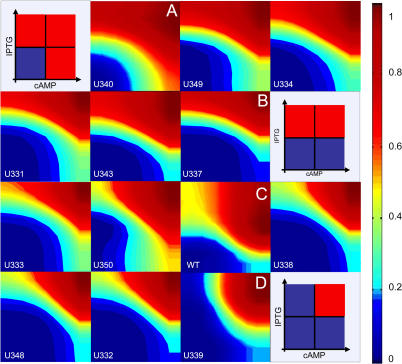
Input Functions of *lac* CRR Variants The input functions are ordered from top left to bottom right to range from those that most resemble OR gates to those that most resemble AND gates. (A) The most OR-like input function. (B) Input function resembles a single-input switch that responds only to IPTG and not to cAMP (note the elevated plateau III compared to the wild-type). (C) Wild-type input function. (D) The most AND-like input function, whose main difference from wild type is a lower plateau III.

We find diverse input functions in our library. Two examples, as well as the CRIF of the wild-type promoter region, are shown in Figure 4. All 12 input functions are shown in Figure 3 and Table 1. Several variants showed an OR-like CRIF ( Figure 3D, 3E, and 3F), with a high plateau when at least one inducer is present and a low plateau when both are absent. Other CRR variants showed a more AND-like CRIF ( Figure 3G, 3H, and 3I), in which plateau III of the wild-type CRR is significantly lowered. The AND-like CRIF has a high plateau when both inducers are present and low expression otherwise.

**Table 1:**
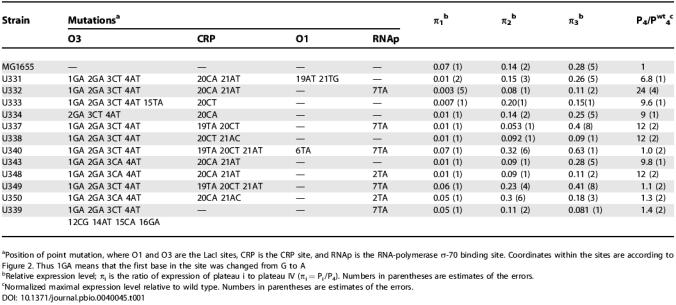
*Lac* Promoter Variants and Their CRIFs

As shown in Figure 4, the CRR variants were all similar to AND gates, OR gates, or to functions that are intermediate between AND and OR gates. Some of the input functions resemble single-input switches rather than AND/OR gates, in the sense that the response to one input is much stronger than to the other input. An example is strain U337 ( Figure 4) that has a stronger response to IPTG than to cAMP.

### Mathematical Model

We used a model for the *lac* CRIF based on the equilibrium binding of RNAp and the two regulators, CRP and LacI, to the *lac* promoter region. In our previous study, this model was found to describe well the wild-type CRIF [ 8]. The mathematical model describes the system at the level of effective binding affinities of the regulators. The three parameters in the model that define the interactions of CRP, LacI, and RNAp with their DNA sites are denoted *d, c,* and *a.* The mutant variants in this study correspond to input functions in which these affinity parameters are varied with respect to the wild-type input function.

### Parameter Space and Phenotype Space

**Figure 5:**
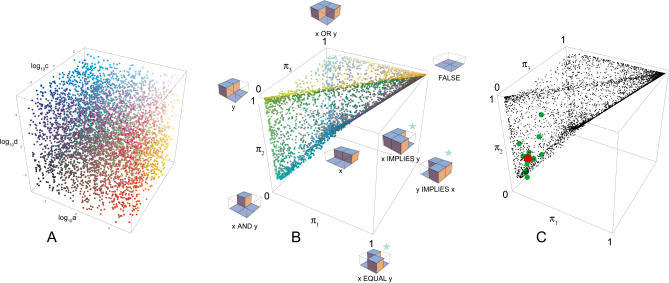
Parameter Space and Phenotype Space of the *lac* CRIFs (A) Parameter space is the space of the three parameters *a, c,* and *d* that describe the relative binding affinity of RNAp, LacI, and CRP to their sites in the *lac* CRR; 5,000 points log-uniformly distributed in this space are shown. (B) Phenotype space, whose axes correspond to the ratio of plateaus I, II, III to plateau IV in the input function (see Figure 3C for definition of these plateaus). The phenotypes of the points in (A) are shown; corresponding points in parameter and phenotype space have the same color. Also plotted are the input functions that correspond to the eight extreme corners of phenotype space. Input functions marked with a star cannot be reached with point mutations in the regulator binding sites according to the model. In the case of the (1, 1, 1) vertex, all plateaus are equal (either to 1 or 0). We depict this situation by the FALSE gate. (C) Phenotype space: the experimentally observed input functions are indicated in green dots and that of the wild type promoter region in a red dot; black dots are the phenotypes of points shown in (A).

The model allows a convenient description of the range of CRIFs that can be reached by point mutations in the CRR. The three parameters that define the interactions of the regulators and RNAp with their DNA sites, *a, c,* and *d* can be used to define a 3-D parameter space of possible CRR variants ( Figure 5A).

Each point in this parameter or “genotype” space corresponds to a specific CRIF “phenotype.” To describe the space of phenotypes, note that each CRIF can be described by the ratios of the three plateaus (plateaus I, II, and III defined in Figure 3) to the fully induced plateau (Plateau IV): π _1_ is the ratio of expression with no inducers to expression with both saturating inducers, and π _2_ and π _3_ are the ratios of expression with only one inducer (IPTG or cAMP, respectively) to expression with both. This defines a 3-D phenotype space or CRIF space ( Figure 5B). Pure logic gates lie at the vertices of this space. For example, pure AND gates have coordinates ([π _1,_ π _2,_ π _3_] = [0,0,0]), pure OR gates have coordinates (0,1,1), and “dysfunctional gates” (in which all plateaus are equal either to 1 or 0) have coordinates (1,1,1), as shown in Figure 5B. Figures 5A and 5B show how uniformly distributed points in parameter space result in a nonuniform distribution of phenotypes in CRIF space. The phenotypes are all confined to a restricted region of CRIF space. This indicates that some CRIFs cannot be reached by point mutations in the CRR. The unreachable CRIFs include the EQUAL gate, in which expression occurs when neither, or both, inducers are present, but not if only one is present. An additional “forbidden” CRIF is an XOR gate, where expression occurs when only one, but not both, inducers are present. All ten forbidden forms are noted in Figure 1.

In contrast to forbidden CRIFs, some CRIF forms lie in dense regions of the design space and, thus, can be readily reached by point mutations in the CRR. These functions include AND gates, OR gates, and single-input switches.

The CRR variants in the present study are represented in phenotype space in Figure 5C. The characterized variants form a cloud in phenotype space around the wild type. The mutations in our study thus appear to form phenotypes in the vicinity of the wild type. Additional mutations should allow access to variants that are more broadly distributed in parameter space.

## Discussion

The effect of point mutations on the input function of a promote region was studied. We found that a few point mutations can change the input function significantly, resulting in AND-like gates, OR-like gates, and single-input switches. The observed CRIF variants can be explained in a unified way by means of a mathematical model. The model explains which gates cannot be reached by point mutations in the regulator sites.

The mathematical model allows depiction of the mapping between parameter space and phenotype space (or CRIF space) ( Figure 5A and 5B). Note that the density of points in phenotype space is not uniform. Some functions seem to be easier to find in a random mutational walk in parameter space than others. Sparse regions of CRIF space correspond to input functions that are very plastic with respect to mutations: small changes in parameters can carry them far from their original phenotype. (This can be quantified by measures such as the Jacobian of the transformation of model parameters to phenotypes.) In particular, the input function of the wild-type *lac* promoter region is in a rather sparse region in CRIF space. To preserve the wild-type CRIF, mutants must continually be selected against; genetic drift would tend to shift the input function away from its present position. In contrast, a pure AND gate, close to the lower left corner of the space, would be more robust to mutations [ 33] and remain AND-like despite significant changes in binding-site parameters.

The wild-type CRIF appears to be easily changed by mutations into new functions. It is plastic in the sense that many mutations do not ruin the input function completely, but rather result in a new computation, a new way to integrate the inputs. A CRIF that can access potentially useful computations with few mutations can readily adapt in case the environmental conditions change [ 1]. Not all new computations can be learned, however, with point mutations in the *cis*-regulatory sites of this promoter. The range of functions that can be reached by simple point mutations is strongly constrained by the structural form of the CRR and its input regulators. For example, input functions in which plateau IV is lower than plateau I, II, or III cannot be reached. Similarly, input functions in which plateau I is higher than any other plateau cannot be reached. The set of forbidden functions include NAND, NOR, XOR, and EQUAL gates, and others (ten of the 16 possible two-input logic functions are forbidden; Figure 1 **).** These forbidden input functions might conceivably be useful in some environments. They may be reached by rearrangements of the promoter region [ 7] or by new protein–protein interactions. For example, making the CRP site overlap the RNAp site can turn the activator CRP into a repressor [ 34] and allow access to additional input functions.

The present approach can also be used to construct and characterize new input functions. New input functions are useful for the design of synthetic gene circuits made of well-characterized transcription factors [ 24, 35– 50]. Most synthetic circuits built so far have promoters with only a single-input regulator. Addition of multi-input functions [ 5, 51] could significantly strengthen the computational power of synthetic gene circuits and mimic real biological design [ 52, 53]. One limitation is our current lack of understanding of the precise mapping between the DNA sequence of a binding site and the model parameters that describe its effective in vivo affinity. For example, we find that some of the changes in the CRIFs do not correlate in a simple way with the distance of the mutated binding sites from their consensus sequences. That is, in some cases a mutation moved a site closer to its consensus sequence, whereas the model affinity parameter was predicted to be lower for the mutated CRR than for wild type (unpublished data). Indeed, the in vivo affinity and efficacy of a regulator may depend on the sequence context outside of its site. This means that in some cases we may not be able to fully predict in vivo parameters based solely on the DNA sequence of the CRR, requiring an empirical search similar to the present study.

Our main finding is that a few mutations can change an input function significantly, resulting in AND-like gates, OR-like gates, and single-input switches. The present study thus relates to the question of how input functions are shaped by evolutionary selection. This question may also be further studied experimentally [ 22, 23, 25, 54, 55] by evolving bacteria in defined environments that favor different input functions. It would also be interesting to study how input functions vary between species that live in different environments. The present experimental and theoretical approach could be readily extended to study plasticity in other gene systems.

## Materials and Methods

### Plasmids and strains

Promoter activity was measured using low-copy plasmids [ 8] that report for transcription rate of a fast-folding GFP reporter from the CRR of interest. The wild-type *lac* CRR of E. coli K12 strain MG1655 was used as a basis for mutations. Variants of the CRR were generated by custom synthesis (BaseClear, Leiden, The Netherlands) of a 113-bp DNA fragment with the sequence (genomic coordinates 365438–365669): AAW TGTGAGC GCAACGCAAT TAATGTGAGT TAGCTCACWW H TTAGGCACC CCAGGCTWTA CACTTTATGC TTCCGGCTCG W ATGTTGTGT GGAATTGTGA GCGGATAACA ATT, where W at positions 3, 39, 40, 57, and 81 is A or T with equal probability and H at positions 41 is A, T, or C with equal probability. The CRR library was cloned into pU66 [ 8] and transformed into MG1655. Colonies were isolated and the CRR was fully sequenced. One of the CRR variants (U339) was synthesized (GenScript, Piscataway, New Jersey, United States) to also have the O3 sequence replaced by the O1 sequence. Reporter strains are listed in Table 1.

### Culture and growth conditions

Cultures (1 ml) inoculated from single colonies were grown for 16 h in M9C defined medium (M9, 2 mg/ml glucose, 1 mM MgSO _4_, 0.1 mM MgCl _2_, 25 μg/ml kanamycin) at 37 °C with shaking at 250 rpm. To map each CRIF, the cultures were diluted to OD _600_ = 0.003 into M9C with different concentrations of cAMP (0–20 mM, Sigma, St. Louis, Missouri, United States) and IPTG (0–200 μM), at a final volume of 150 μl per well in a flat-bottomed 96-well plate (Sarstedt, Beaumont Leys, United Kingdom). The cultures were covered with 100 μl of mineral oil (Sigma) to prevent evaporation and grown for about 18 h in a WallacVictor2 multiwell fluorimeter (PerkinElmer, Wellesley, California, United States) at 37 °C, set with an automatically repeating protocol of shaking and OD _600_ and fluorescence readings [ 8]. Time between repeated measurements was 6 min. In order to correct for the differences in growth rates (especially due to the different concentrations of cAMP), background fluorescence at a given OD was determined from the fluorescence of cells bearing a promoterless GFP vector at the same OD and at the same cAMP concentrations (total of 12 different control conditions. IPTG was not found to have a large effect on the cell growth rate, data available on request). Cells growing on glucose, with saturating external cAMP, and cells growing on glycerol (high endogenous cAMP), without exogenous cAMP, show similar *lac* promoter activity and growth rates. The rate of GFP production, divided by the OD at midexponential growth, provided a measure of the promoter activity: PA = dGFP/dt/OD [ 8]. Note that promoter activity measurement takes dilution by growth into account because if GFP is produced at rate β per cell per unit time, and there are *N*(t) cells then dGFP/dt = β *N*(t). At all conditions, the promoter activity achieved an approximately constant value during about two cell cycles in midexponential growth. We computed the promoter activity in each of the 88 growth conditions by an average of the promoter activity over these two cell cycles, resulting in the CRIF map. Day-to-day variability in fluorescence and OD data gathered from the instruments was about 10% (both for GFP fluorescence and absorbance) [ 56]. The relative error in the promoter activity measurement is about 15%. Each of the variants in our study was mapped in the same conditions and in the same strain as all other variants. Hence, the changes in GFP expression result from the mutations in the promoter. The wild-type *lac* system is intact on the chromosome and identical for all variants.

In our previous study [ 8], we reported a comparison of the present GFP-reporter plasmid and a direct chromosomal enzyme production assay using a colorimetric substrate to assay the production of LacZ from its endogenous locus. The *lac* system has classically been studied by using such colorimetric assays for the *lacZ* gene product. The present GFP-reporter plasmid measurement is different in several ways from assays of enzyme activity from the chromosomally encoded operon: (i) the low-copy plasmid (pSC101 origin) introduces several extra copies of the promoter region, thus potentially titrating out LacI; (ii) the promoter region on the plasmid lacks the O2 binding site (+411 in the lacZ coding region), a site whose absence makes shutoff of the promoter about 5-fold weaker; and (iii) the plasmid DNA may be harder to loop, reducing repression strength. The previous experiments [ 8] with the colorimetric assay, using accurate time-resolved ONPG absorbance measurements, indicated that the input functions found by the two methods are qualitatively similar with four plateaus and four threshold levels. However, some of the plateaus were deeper in the ONPG assay. This difference presumably reflects the above-mentioned plasmid effects.

We also measured cell–cell variability in GFP expression using flow cytometry with a narrow gate on side- and forward-scatter (Figure SOM 1A in Protocol S1). We found that in the present conditions the GFP distributions were single peaked, and few all-none effects [ 57, 58] were discerned (Figure SOM 1B in Protocol S1).

### Mathematical model

Promoter activity was modeled based on equilibrium binding of the regulators CRP and LacI as described in [ 8]. The promoter activity is:



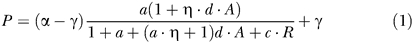



where CRP activity (fraction of CRP bound to cAMP) is *A* = [CRP–cAMP]/[CRP _T_] = *X ^n^/ *( *1 + X ^n^*), in which *X* = [cAMP]/K _cAMP_ is cAMP concentration in units of its dissociation constant for CRP. Cooperativity is described by the Hill coefficient *n.* Similarly, the fraction of LacI not bound to IPTG is *R* = [LacI-free]/[LacI _T_] = *1/*( *1 + Y ^m^*), with *Y* = [IPTG]/K _IPTG_, where *m* is the Hill coefficient. Three parameters, *a, c,* and *d* define the binding affinity of the regulators RNAp to their sites: *a* = [RNAp]/K _P_ is RNAp concentration in units of its dissociation constant to a free promoter when cAMP–CRP is not bound to the CRR, *c* = [LacI]/K _R_ is LacI concentration in units of its dissociation constants to its site, and *d* = [CRP]/K _C_, is CRP concentration in units of the dissociation constant for binding to its site. The stabilization of RNAp binding by CRP is given by the ratio of its affinity without and with cAMP–CRP binding: η = K _P_/K _CP_ (using the notation of [ 8] Equation 10, η = *b/a*. Note the typo with 2 *b* instead of *b* in the corresponding equation in [ 8]). Finally, α and γ are the maximal and basal transcription rates. We note that the present experimental data do not appear to be sufficient to obtain unique fits to all of the model parameters, without further measurements (such as direct estimates of the parameter η).

## Supporting Information

Protocol S1 Plasticity of the CRIF of a Gene (171 KB PDF)

## Abbreviations

CRIF: *cis*-regulatory input function
CRP: cAMP receptor protein
CRR: *cis*-regulatory region
GFP: green fluorescent protein
IPTG: isopropyl-D-thiogalactoside
RNAp: RNA polymerase
