# Supplementary material for: Plasticity of the cis-Regulatory Input Function of a Gene
Source: PLoS Biol. 2006 Mar 28;4(4):e45. doi: 10.1371/journal.pbio.0040045 (PMC1413569; doi:10.1371/journal.pbio.0040045)
Supplement: Supplementary file 1 [file pbio.0040045.sd001.pdf]

Plasticity of the cis-regulatory input function of a gene - Supporting Information

A. E. Mayo, Y. Setty, S. Chalamish, A. Zaslaver and U. Alon

We begin this SOM with results on the lac promoter activity in individual cells. We continue with a discussion and clarifying remarks on the mathematical model used to analyze the CRIFs found in this study.

Distribution of lac promoter activity in individual cells

Distribution of lac promoter activity in individual cells induced with 96 combinations of cAMP (0-20 mM) and IPTG (0-200 μM), was measured by flow cytometry (FACS) with a narrow gate on side and forward scatter (fig. SOM 1 a). The narrow gating was aimed at reducing the variation in cell sizes used for the measurement. The gating was performed using custom Mathematica software on the FACS output data. Cell-cell variability in GFP expression was measured. The map is shown in fig. SOM 1 b. Color indicates the mean fluorescence. Under the present conditions the GFP distributions were mostly single peaked and few broad distributions were found. The four plateaus, four thresholds and the diagonal are clearly discernable. Note that under some conditions, all-none effects and broad distributions are possible in this system (1, 2).

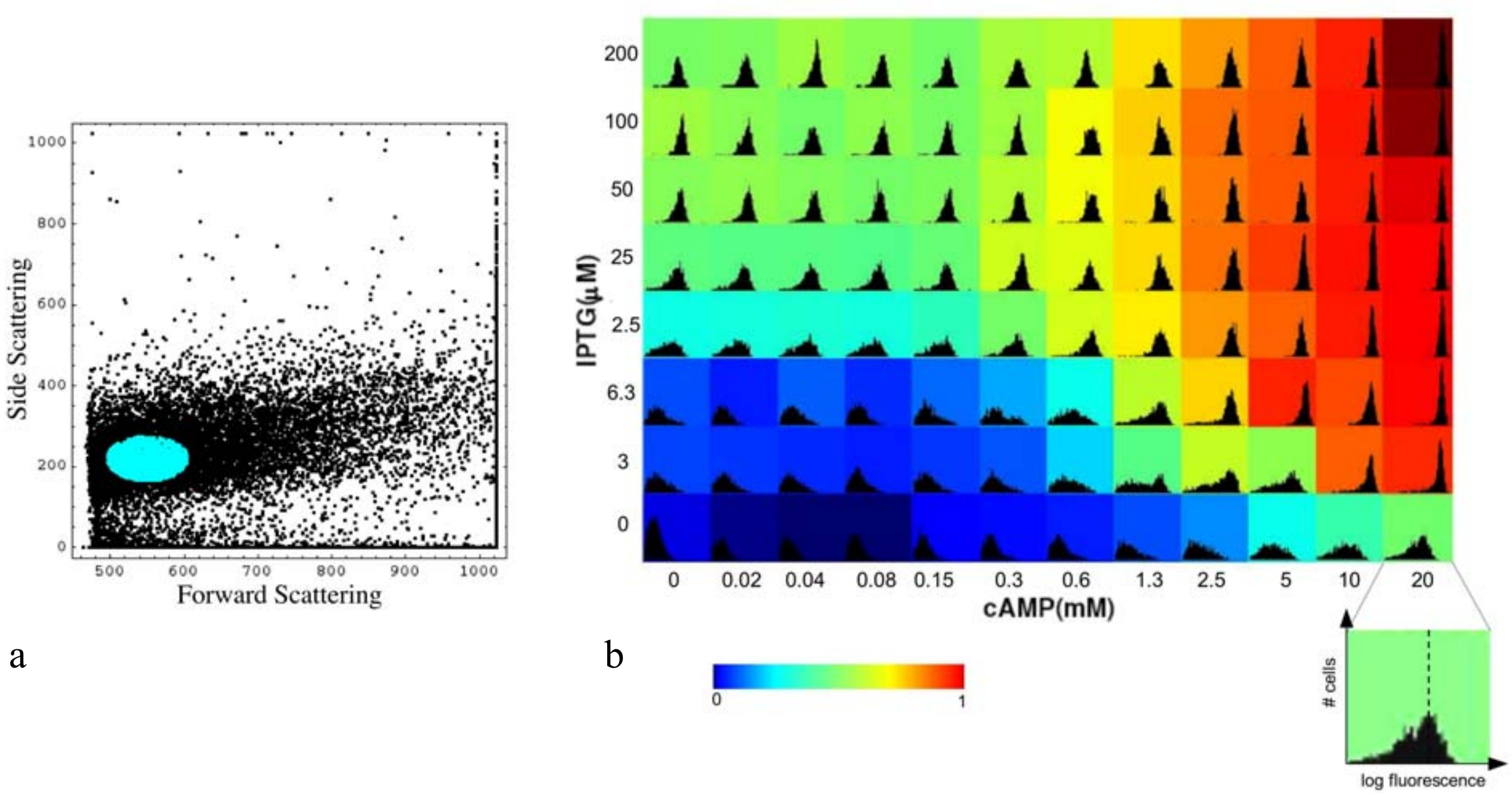

Fig. SOM 1. Distribution of lac promoter activity in individual cells. a) Gate used in the flow cytometry experiment. The region of forward and side scattering that was chosen for measurement of GFP expression is shaded in cyan. b) Map of cell-cell variability in GFP expression in 96 combinations of the inducers.

Mathematical model

The analysis starts with the expression for the promoter activity as derived form the model Setty et al (3):

$$PA = (\alpha - \gamma)\frac{a(1 + \eta d\mathrm{A})}{1 + a + (a\eta + 1)d\mathrm{A} + c\mathrm{R}} + \gamma .$$

Here A= [CRP-cAMP]/[CRP_T] = $X^n/(1 + X^n)$, in which X =[cAMP]/K_{cAMP} is cAMP concentration in units of its dissociation constant for CRP. Cooperativity is described by the Hill coefficient *n*. Similarly, R=[LacI-free] / [$LacI_T$] = $1/(1 + Y^m)$, with Y = [IPTG]/K_{IPTG}, where *m* is the Hill coefficient. The high (low) cAMP limit is obtained when $\mathrm{A} \to 1$ $(\mathrm{A} \to 0)$. Similarly, the high (low) IPTG limit is obtained when $\mathrm{R} \to 0$ $(\mathrm{R} \to 1)$. The three parameters, a, c and d, define the binding affinity of the regulators and RNA-polymerase (RNAp) to their sites: a=[P]/K_P is RNAp concentration in units of its dissociation constant to a free promoter when cAMP-CRP is not bound to the CRR, c=[R_T]/K_R designates LacI concentration in units of its dissociation constants to its site. d = [CRP_T]/K_C, is CRP concentration in units of the dissociation constant for binding to its site. The stabilization of RNAp by CRP is given by the ratio of its affinity without and with cAMP-CRP binding: $\eta = K_P/K_{CP}$. Finally, α and γ are the maximal and basal transcription rates. Note that this model does not explicitly take into account the effect of DNA looping. However, previous work showed that the effect of looping could be implicitly described by a single parameter for the repressor for the purposes of modeling the lac CRIF (4). This model was found to describe the data to within experimental error. Adding additional terms to the model did not significantly improve the fit.

The four plateaus, which characterize a CRIF, are obtained by taking the four limits

$$P_1 = \lim_{\substack{\mathrm{A} \to 0 \\ \mathrm{R} \to 1}} PA = (\alpha - \gamma)\frac{a}{a + c + 1} + \gamma,$$

$$P_2 = \lim_{\substack{\mathrm{A} \to 1 \\ \mathrm{R} \to 1}} PA = (\alpha - \gamma)\frac{a}{a + \frac{c + d + 1}{\eta d + 1}} + \gamma,$$

$$P_3 = \lim_{\substack{\mathrm{A} \to 0 \\ \mathrm{R} \to 0}} PA = (\alpha - \gamma)\frac{a}{a + 1} + \gamma,$$

$$P_4 = \lim_{\substack{\mathrm{A} \to 1 \\ \mathrm{R} \to 0}} PA = (\alpha - \gamma)\frac{a}{a + \frac{d + 1}{\eta d + 1}} + \gamma.$$

It is important to note that the four plateaus are independent of the cooperativity parameters *n* and *m* as well as the inducer-proteins binding constants, K_{cAMP}, and K_{IPTG}.

The mapping from the 3D *parameter space* to the 3D *design space* is done by the function

$$f_{\{\alpha,\gamma,\eta\}}:\mathbb{R}^3 \to \mathbb{R}^3,$$
$$f_{\{\alpha,\gamma,\eta\}}(a,c,d) = \{\pi_1,\pi_2,\pi_3\}, \qquad \pi_i = P_i/P_4.$$

Assuming that CRP always serves as an activator (e.g. $\eta > 1$), a quick check reveals that $\pi_1 \le \{\pi_2,\ \pi_3\} \le 1$. This simple observation indicates that the model predicts that CRIFs that violate this inequality (i.e. XOR, EQUAL, NAND, etc.) cannot be reached from the wild type *lac* CRIF by a continues change of the parameters a, c, or d (which corresponds to a gradual change of the *lac* CRR by a series of point mutations). Using the definition for *f* above we can understand how the parameters control the logic of the gate encoded on the CRR. The CRIFs that have Boolean-like structures are attained in the limits in which the parameters are extreme. For example, in the limit $c \to 0$ (i.e. the CRR can no longer be regulated by the LacI repressor) we obtain a single input switch – an X switch (coordinate (ε, 1, ε) in Fig 5b in the article; $0 \le \varepsilon < 1$). Similarly, in the limit $d \to 0$ (i.e. CRP completely fails to activate), we get the second type of a single input switch – a Y switch (coordinate (ε, ε, 1) in Fig 5b). A CRIF with a more complicated structure can be obtained by a simultaneous change of the parameters. For example, a CRIF with a Boolean-like OR logic, is obtained by a combination of the parameters such that $1 \ll a \ll c < d$. The rational behind this choice of parameters is that by having an efficient binding of the polymerase (high *a*) the output of the gate (in all conditions) is elevated. In order to compensate for the higher expression from plateau I (low IPTG/ low cAMP), the binding of the repressor is increased (high *c*). This however lowers the expression from plateau II (low IPTG/high cAMP) as well. In order to compensate for this the binding of CRP is increased (high *d*). A CRIF with a Boolean AND logic can be obtained by a combination of parameters such that $a < 1 < d < c$. Here the rational is that by having poor binding of the polymerase (low a) the total output of the gate is decreased. In order to compensate for this the binding of CRP is increased so that it serves as a better activator (higher *d*), which means that the expression from plateau IV (high IPTG/high cAMP) as well as from plateau II (low IPTG/high cAMP) is increased. In order to compensate for the elevated expression from plateau II the binding of the repressor is increased (higher *c*). Other logic functions, intermediate between AND, OR, and single input switches, can be designed using similar reasoning.

1. Novick, A. & Weiner, M. (1957) *Proc. Natl. Acad. Sci.* **43,** 553-566.
2. Ozbudak, E. M., Thattai, M., Lim, H. N., Shraiman, B. I. & Van Oudenaarden, A. (2004) *Nature* **427,** 737-40.
3. Setty, Y., Mayo, A. E., Surette, M. G. & Alon, U. (2003) *Proc Natl Acad Sci U S A* **100,** 7702-7.
4. Eismann, E. R. & Muller-Hill, B. (1990) *J Mol Biol* **213,** 763-75.
